# A New Day for Global Environmental Health

**DOI:** 10.1289/ehp.11322

**Published:** 2008-04

**Authors:** William A. Suk

**Affiliations:** National Institute of Environmental Health Sciences, National Institutes of Health Department of Health and Human Services, Research Triangle Park, North Carolina, E-mail: suk@niehs.nih.gov

This April marks the celebration of two significant global events relevant to the mission of the National Institute of Environmental Health Sciences (NIEHS). The World Health Organization (WHO) has designated 7 April to be World Health Day, and the theme of this year’s event is protecting health from climate change (WHO 2008). On 22 April an estimated 1 billion people around the world will celebrate Earth Day, which this year focuses on solving issues of climate change (Earth Day Network 2008). It is not by accident that the planners of both of these events chose climate change as the lens through which to focus our attention; many regard climate change as the greatest global challenge we have ever faced. Just as climate change may represent the intersection of human health and the environment in its worst iteration, global environmental health may represent our greatest opportunity to understand and ultimately affect the outcome of this relationship. The NIEHS, with a public health mandate, a strong history of global cooperation on environmental health problems, and a research vision aimed at solving the puzzles of environmental disease, is uniquely poised at the front lines of global environmental health.

Although not always defined in a global context, it can be argued—particularly in today’s modern society of fluid borders, global transport and economies, competition for natural resources, and transboundary pollution—that essentially all environmental health is global. Examples such as lead in toys that are made in one country and sold in another, food that is grown or harvested locally and shipped to tables worldwide, and pollution that is generated in one place and migrates through air or water with far-reaching and often unexpected consequences show us that our perspective on environmental health must be broad. For the NIEHS, this is a longstanding and ongoing approach.

As a public health institute, the NIEHS has always had a commitment to the goals of protecting and improving global health. Former NIEHS director David Rall served as the U.S. coordinator of cooperative environmental health programs between the United States and the former U.S.S.R. (Union of Soviet Socialist Republics), the United Kingdom, Egypt, Japan, the People’s Republic of China, Taiwan, Italy, Finland, and Spain. In 1975, the NIEHS was named by the WHO as a Collaborating Center for Environmental Health Effects. The NIEHS has also participated for 27 years in a cooperative agreement with the International Programme on Chemical Safety, through which the institute has helped to provide scientific leadership and expertise to efforts to protect public health worldwide from effects of toxic chemicals. Similarly, NIEHS scientists have historically lent their expertise to international organizations formulating scientific consensus such as the International Agency for Research on Cancer, the International Labor Organization, and the United Nations Environment Program.

However, the NIEHS investment in global environmental health goes well beyond shared expertise. Over the years the institute has funded and collaborated in wide-ranging scientific endeavors in partnership with scientists and scientific organizations in other countries, and continues to invest significant resources in such projects. A recent analysis of this investment shows that from 2005 to 2007, the NIEHS was engaged in 57 projects in 37 different countries (Drew et al. 2008). These projects range from investigating the effects of heavy metals on children’s development along the U.S.–Mexico border, to elucidating the role of aflatoxin in inducing liver cancer in people in rural China, to following the long-term disease consequences for women exposed to dioxin in Italy. Similarly, NIEHS intramural scientists are also involved in a number of collaborations with researchers in foreign countries, such as investigating possible genetic risks for preeclampsia in Norway and genetic mechanisms of host response to respiratory syncytial virus (RSV) infection in infants in Argentina.

The justification for investing in global environmental research is simple: You must go where the exposures are. It is an unfortunate truism that the greatest exposures also hold the greatest opportunity for understanding how factors in our environment interact with the human body to cause and exacerbate disease. The more we understand these most basic interactions, the more hope we can have for finding ways to prevent and treat adverse environmental effects and improve human health. In many cases, the greatest exposures are occurring in countries outside the United States; however, the level of resources and scientific expertise enjoyed by our nation demands that we take a stewardship role in global environmental health research. The NIEHS is committed to this goal in a number of ways: through direct funding of research projects, through scientific collaboration, and through environmental health science training and capacity building.

Almost from its inception, the NIEHS began welcoming foreign researchers and students to its North Carolina campus to train with the best scientists our public health system had to offer. To date, thousands of visiting fellows and other foreign trainees have taken advantage of the expertise and facilities of the NIEHS to learn cutting-edge science, improve their knowledge and skills, and bring these abilities to bear on pressing environmental health problems around the world. Currently the NIEHS supports approximately 100 international fellows each year in an effort to build global scientific capacity, as well as to create networks of collaborators who can leverage the power of multiple minds and perspectives on environmental health issues. The NIEHS also supports a number of other activities that either directly or indirectly build environmental health science capacity in other countries such as scientific conferences and meetings, including two in 2006 specifically targeted at identifying global environmental health issues and exploring research partnerships (NIEHS 2007). Perhaps the most visible aspect of this capacity building is the NIEHS’s publication of *Environmental Health Perspectives*, which through its policy of Open Access and commitment to dissemination of research and information to the developing world, provides a global foundation of knowledge on environmental health science.

It is from this fertile background that the vision of the next phase of the NIEHS’s commitment to global environmental health is emerging. The first crystallization of this vision is an NIEHS website that will serve as a portal to information on the institute’s investment in this area (NIEHS 2008). Initially, this new site will catalog and describe ongoing NIEHS activities in the categories of research collaborations, training, outreach and capacity building, and international scientific service. Our goal for this new website is to enable us to leverage the research already being done by connecting interested parties around the world looking at the same or similar problems. The site will also provide more visibility to NIEHS funding mechanisms for international research and training programs, and it will provide a dedicated venue for communicating the outcomes and value of this investment to the public. In the long term, we envision expanding this website into a much broader and more robust networking space that will include online collaboration tools and interactive research technologies with the capacity to connect scientists around the world in real time to enhance real progress.

The NIEHS is strongly committed to continuing its tradition of active engagement and leadership in global environmental health. In fact, the NIEHS 2006–2011 Strategic Plan (NIEHS 2006) specifically identifies global environmental health research, capacity building, training, and partnerships as a priority for our institute. As the celebrations of World Health Day and Earth Day this month bring into sharp relief, there has never been a greater need or a better time for the NIEHS to reaffirm our efforts toward these goals.

## Figures and Tables

**Figure:**
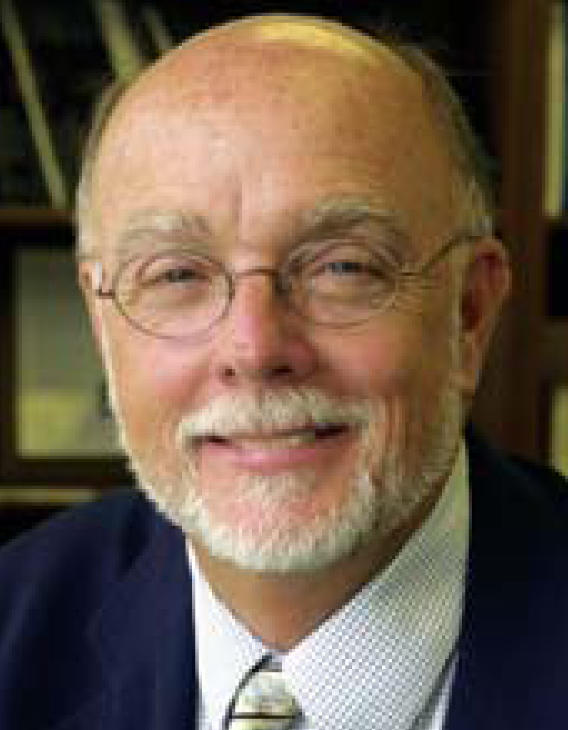
William A. Suk
